# Long-term prediction of changes in health status, frailty, nursing care and mortality in community-dwelling senior citizens - results from the longitudinal urban cohort ageing study (LUCAS)

**DOI:** 10.1186/1471-2318-14-141

**Published:** 2014-12-19

**Authors:** Ulrike Dapp, Christoph E Minder, Jennifer Anders, Stefan Golgert, Wolfgang von Renteln-Kruse

**Affiliations:** Albertinen-Haus Geriatrics Center, Scientific Department, University of Hamburg, Sellhopsweg 18-22, D-22459 Hamburg, Germany; Horten Zentrum, University of Zürich, Postfach Nord, Zürich, Switzerland

**Keywords:** Population-based screening, Self-report, Functional ability, Functional decline, Frailty, Elderly persons

## Abstract

**Background:**

The detection of incipient functional decline in elderly persons is not an easy task. Here, we propose the self-reporting Functional Ability Index (FA index) suitable to screen functional competence in senior citizens in the community setting. Its prognostic validity was investigated in the Longitudinal Urban Cohort Ageing Study (LUCAS).

**Methods:**

This index is based equally on both, resources and risks/functional restrictions which precede ADL limitations. Since 2001, the FA index was tested in the LUCAS cohort without any ADL restrictions at baseline (n = 1,679), and followed up by repeated questionnaires in Hamburg, Germany.

**Results:**

Applying the index, 1,022 LUCAS participants were initially classified as Robust (60.9%), 220 as postRobust (13.1%), 172 as preFrail (10.2%) and 265 as Frail (15.8%). This classification correlated with self-reported health, chronic pain and depressive mood (rank correlations 0.42, 0.26, 0.21; all p < .0001). Survival analyses showed significant differences between these classes as determined by the FA index: the initially Robust survived longest, the Frail shortest (p < .0001). Analyses of the time to need of nursing care revealed similar results. Significant differences persisted after adjustment for age, sex and self-reported health.

**Conclusions:**

Disability free lifetime and its development over time are important topics in public health. In this context, the FA index presented here provides answers to two questions. First, how to screen the heterogeneous population of community-dwelling senior citizens, i.e. for their functional ability/competence, and second, how far away they are from disability/dependency. Furthermore, the index provides a tool to address the urgent question whether incipient functional decline/incipient frailty can be recognized early to be influenced positively.

The FA index predicted change in functional status, future need of nursing care, and mortality in an unselected population of community-dwelling seniors. It implies an operational specification of the classification into Robust, postRobust, preFrail and Frail. Based on a self-administered questionnaire, the FA index allows easy screening of elderly persons for declining functional competence. Thereby, incipient functional decline is recognized, e.g. in GPs’ practices and senior community health centers, to initiate early appropriate preventive action.

**Electronic supplementary material:**

The online version of this article (doi:10.1186/1471-2318-14-141) contains supplementary material, which is available to authorized users.

## Background

Providing health care and social services for the growing number of elderly persons under tight budgetary constraints is challenging [1]. Comprehensive geriatric assessment may ease that burden in clinical settings [2] but population-based screening tools to distinguish between persons who are robust, those at risk of functional decline and who are functionally limited are still lacking [3].

Research has identified precursor states to failing health and functional deterioration with grades of derangement (prefrail), functional decline (frail) and persons who are already severely functionally limited (disabled). The phenotypes prefrailty and frailty are generally described as reduced resistance to stressors and increased vulnerability to adverse outcomes; a condition probably caused by cumulative declines across multiple physiologic systems [4–6]. Despite a lack of unanimity on how to define frailty [7–10], there is growing consensus that frailty is distinct from disability and comorbidity [11]. Frailty is seen as a dynamic transition that may be slowed or reversed [12] to prevent disability, institutionalization and mortality [6].

Several frailty indices include medical and functional data, comprising many symptoms, diagnoses and impaired function which require clinical assessments that are based on face-to-face contacts and performance tests [8, 13–16]. In fact, “frailty is the most problematic expression of population ageing” [17] and early detection of pre-clinical stages, adequate preventive measures for persons at risk of becoming frail, and their appropriate geriatric treatment are urgently needed [17, 18].

Therefore, we will not further pursue clinical indices describing frailty in advanced stages. Rather, we discuss self-reported frailty indices suitable for screening the elderly population in the community setting. Examples of such non-clinical frailty indices are the Tilburg Frailty Indicator [19], the Sherbrooke Postal Questionnaire [20] and the Groningen Frailty Indicator (Steverink et al. cited in 21). Two of these instruments [20, 21] include ADL limitations. One study found insufficient predictive validity for all three indices and concluded that longitudinal research into the psychometric properties was needed [21]. The Tokyo Metropolitan Institute of Gerontology Index of Competence (TMIG) focuses on self-reported activities in the environmental domain, and measures higher-level functional capacity such as instrumental self-maintenance, intellectual activity and social role [22]. This is important with ageing in place (rather than in institutions).

The Functional Ability Index (FA index) investigated here is based on self-report. The eleven questions in the questionnaire underlying this index can be completed by elderly persons themselves. Contrary to other indices [20, 21], but according to Frieds’ frailty phenotype [4, 11] we did exclude disability. We also included specific questions about daily life to address functional decline as early as possible. These new questions integrate high order functions such as outdoor mobility and social interaction. Subsequently, we term these items ‘resources’. Contrary to the indicators described in [19–22] the resulting index is based equally on both, functional risk factors and resources. As will be shown, this permits distinguishing between fully robust persons and those not fully robust, but better off than prefrail, i.e. very early on in functional decline.

We investigated the predictive power of the FA index for changes in health status, development of need of nursing care, and mortality over a period of nearly eight years (93 months).

Our research questions were:

1. Does the FA index distinguish between robust, prefrail and frail persons?
2. Does the functional classification based on the FA index correlate with other measures of health, e.g. self-reported health?
3. Does the FA index predict the need of nursing care and death over time?
4. The FA index defines functional classes. What were predominant transition paths between these functional classes in the period 2001/02 to 2007/08?

## Methods

This study was performed as part of the LUCAS Consortium in Hamburg, Germany, an interdisciplinary research cooperation studying various aspects of aging in an urban population in different settings [23]. The LUCAS core project is a long-term cohort of elderly persons recruited between October 2000 and July 2001 via 21 general practitioners (GPs) from the Hamburg metropolitan area. The GPs provided lists of all their patients aged 60 years and older and excluded patients in need of daily human help or those entitled to nursing care according to German law (9.0%), with cognitive impairments (equivalent to a Mini Mental Status score of ≤24; 9.6%), terminal disease (1.4%) or insufficient command of German (1.4%). This recruitment resulted in a quite healthy and robust cohort of elderly persons at baseline (n = 3,326). Participants returned a short baseline questionnaire and their consent form. Extensive data were collected by questionnaire in 2001/2002 (wave 1), 2007/2008 (wave 2), 2009/2010 (wave 3) and 2011/2012 (wave 4). The full details of the study design are described elsewhere [24].

**Figure 1:**
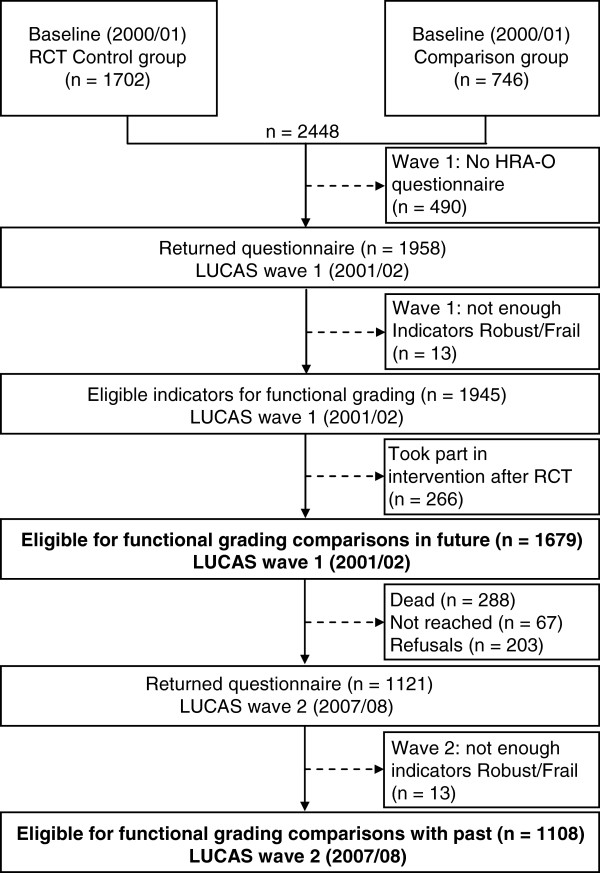
**Flow of study participants up to LUCAS wave 2.**

Initially, the cohort was set up for an RCT of prevention [25, 26]. However, this article is exclusively based on data from the control participants (n = 1,679) who had completed the baseline and wave 1 questionnaires. Of those, 1,108 participated also in wave 2, six years later (Figure 1); contributing 10,450 person years in total.

At wave 1, an extensive questionnaire covering medical and behavioural aspects as well as health measures [27] was distributed including the questions of the FA index (Table 1). Survival status and information on ambulatory nursing care and nursing home admission were obtained from the GPs and double-checked by Hamburg’s Central Registry and the Central Registry of the Statutory Health Insurance (MDK Nord) after 93 months (reference date Sept 30, 2009). Recruitment and data collection were conducted according to the Declaration of Helsinki. Approvals were obtained from the Data Protection Official MDK Nord, the Ethics Committee of the General Medical Council (Ärztekammer), and the Data Protection Official, City of Hamburg.

**Table 1:** **The functional ability index with marker questions**

Frailty risks ^*^/robustness resources ^†^	Marker questions describing (non) frailty Evaluated in Wave 1 (2001/2002)	Answers ^‡^
Risk_1	Have you lost 5 kg (a stone) or more over the past 6 months without trying to do so?	No (0)
		**Yes (1)**
Risk_2	In the past 12 months, have you changed the way to walk 1 kilometer?	No (0)
		**Yes (1)**
Risk_3	In the past 12 months, have you changed the way to climb 10 steps?	No (0)
		**Yes (1)**
Risk_4	In the past 12 months, have you changed the way to get into or out of a car or bus?	No (0)
		**Yes (1)**
Risk_5	Over the past 7 days, how often did you take a walk outside your home or garden for any reason?	**Never or 1–2 days per week (1)**
		3–4 days or 5–7 days per week (0)
Risk_6	During the 12 past months, have you ever fallen to the ground or floor?	No (0)
		**Yes (1)**
Resource_1	Please indicate if you are able to do each of the following activities. If you do not do them, answer as if you tried to do them yourself. Walk 500 meters?	**Yes (1)**
		Yes, with difficulty or with a device or with help from someone or no (0)
Resource_2	Over the past 7 days, how often did you take a walk outside your home or garden for any reason?	Never or 1–2 days per week (0)
		**3**–**4 or 5–7 days per week (1)**
Resource_3	Over the past 7 days, how often did you engage in moderate sport or recreational activities?^§^	Never (0)
		**Yes, 1–2 days or 3–4 or 5–7 days per week (1)**
Resource_4	Over the past 7 days, how often did you engage in strenuous sport or recreational activities?^§^	Never (0)
		**Yes, 1–2 days or 3–4 or 5–7 days per week (1)**
Resource_5	During the past 7 days did you work for pay or as a volunteer?^§^	No (0)
		**Yes (1)**
Resource_6	Do you limit your activities because you are afraid you will fall?	**No (1)**
		Yes (0)

*Frailty Score: Sum over Risk responses 1–6.

^**†**^Robustness Score: Sum over Resource responses 1–6.

^‡^Bold type: Indicative answers are counting one point.

^§^The activity questions Resources 3 to 5 were slightly rephrased for wave 2.

### The functional ability index (FA index)

Both, risk factors and resources were included equally in the FA index (questions see Table 1): First, we operationalized the frailty phenotype “weight loss, slow gait, weakness, exhaustion, reduced physical activity” [4] plus “instability/falls” [28]. The following resources were included: good endurance, frequent outside walking, moderate/strenuous sports or recreation, regular volunteer or paid work, and no limitation of activity due to fear of falling [29], i.e. we modified the Fried criteria. A further modification concerned Fried’s term prefailty: we included the case of zero risks combined with zero to two resources. The resulting FA index includes eleven questions originating from validated instruments [27]. One question, “taking a walk outside …”, has double weight. It counts either as a resource or as a risk, depending on the person’s answer. For further details see Table 1.

Individuals with three to six frailty risks and zero to two resources were classified as Frail. Persons with three to six resources and zero to two risks were classified as Robust. Those persons simultaneously showing characteristics of robustness (3–6 resources) and frailty (3–6 risks) were classified as postRobust. Finally, those simultaneously showing few characteristics of robustness (0–2 resources) and few characteristics of frailty (0–2 risks) were classified as preFrail (Table 2).

**Table 2:** **The functional ability index as a function of the number of risks and resources, numbers (%) at wave 1**

	3 - 6 Robustness score	0 - 2 Robustness score
**0 - 2 Frailty score**	Robust	preFrail: neither Robust nor Frail
	n = 1022 (60.9%)	n = 172 (10.2%)
**3 - 6 Frailty score**	postRobust: both Robust and Frail	Frail
	n = 220 (13.1%)	n = 265 (15.8%)

In order to assure statistical stability in analyses of transitions, we took the two classes postRobust and preFrail together as one single class termed Transient. The FA index was pilot tested in an additional cohort (n = 391) recruited in 2007/2008 [24] and clinically validated by comprehensive assessment [30].

### Statistical analyses

We used Fisher’s exact tests for discrete data, t-tests for age differences and Spearman correlations. Transitions between successive waves were analysed by descriptive means (graphs and tables). For these analyses, missing values were excluded, and remaining numbers were reported. Life table analyses, log rank tests and proportional hazards regression were used for survival and nursing care data. Drop outs were treated as censored.

## Results

### Population description

Table 3 presents demographic and basic health information for all participants and the classes Robust to Frail. None of the 1,679 participants was dependent on nursing care at wave 1. There was an age trend in that the 60.9% Robust at wave 1 were younger than the Frail (t-test, p < .0001). The fraction of women increased from Robust to Frail (Fisher p < .0005). There were significant differences in the prevalence of self-reported poor health, chronic pain and depression between Robust and Frail (Fisher all p < .0005). There was a consistent increase in the percentage of adverse outcomes when moving from Robust to Transient (postRobust + preFrail) and Frail (Table 3, Additional file 1: Table S1).

**Table 3:** **Demographic and selected health characteristics at wave 1**

Characteristics	ALL n = 1679 (100) cases/n (%)	Women n = 1043 (62.1) cases/n (%)	Men n = 636 (37.9) cases/n (%)	Robust n = 1022 (60.9) cases/n (%)	postRobustn = 220 (13.1) cases/n (%)	preFrailn = 172 (10.2) cases/n (%)	Transients ^*^n = 392 (23.3) cases/n (%)	Frail n = 265 (15.8) cases/n (%)
Age – mean, se	72.3 ± 7.5	72.7 ± 7.7	71.6 ± 7.0	70.0 ± 6.2	74.4 ± 7.5	75.2 ± 7.8	74.7 ± 7.6	77.7 ± 7.8
(min., max.)	(61.2-96.8)	(61.4-96.8)	(61.2-91.9)	(61.2-91.9)	(61.7-93.7)	(61.5-94.0)	(61.5-94.0)	(61.4-96.8)
Women	1043/1679(62.1)	1043/1043(100)	0/636(0)	579/1022(56.7)	153/220(69.5)	123/172(71.5)	276/392(70.4)	188/265(70.9)
Living arrangement, alone	588/1638(35.9)	508/1014(50.1)	80/624(12.8)	287/1005(28.6)	91/212(42.9)	69/165(41.8)	160/377(42.4)	141/256(55.1)
State of health fair/poor	510/1651(30.9)	329/1023(32.2)	181/628(28.8)	167/1006(16.6)	95/217(43.8)	74/168(44.0)	169/385(43.9)	174/260(66.9)
Chronic pain^†^	453/1595(28.4)	330/985(33.5)	123/610(20.2)	199/986(20.2)	96/210(45.7)	39/156(25.0)	135/366(36.9)	119/243(49.0)
Depressed^‡^	211/1582(13.3)	141/970(14.5)	70/612(11.4)	91/971(9.4)	34/212(16.0)	25/156(16.0)	59/368(16.0)	61/243(25.1)
Poor vision^§^	422/1658(25.5)	282/1028(27.4)	140/630(22.2)	182/1014(17.9)	70/216(32.4)	57/170(33.5)	127/386(32.9)	113/258(43.8)
No of medication – mean (min., max.)	3.0(0–16)	3.1(0–16)	2.9(0–14)	2.5(0–11)	3.3(0–16)	3.4(0–12)	3.3(0–16)	4.7(0–14)
ADL dependent^||^	33/1633(2.0)	24/1038(2.3)	9/628(1.4)	1/1019(0.1)	1/219(0.5)	9/168(5.4)	10/388(2.6)	22/259(8.5)

*Transients: postRobust + preFrail.

^†^Do you have pain that never completely goes away?

^‡^How much of the time, during the last month, have you felt so down in the dumps that nothing could cheer you up?

Answers: most of the time, all the time.

^§^Would you say your eyesight using both eyes (with your glasses or contact lenses, if you wear them) is:

Answers: fair, poor, I am completely blind.

^||^Cannot take a bath/shower without human assistance.

Nobody with nursing care (exclusion criterion).

### Validity: correlations

At wave 1, Spearman rank correlations of the FA index with age and the health measures self-reported health, pain and depressive mood were 0.3782, 0.4207, 0.2547 and 0.2074 (all p < .0001), and remained stable over six years (data not shown). The numbers of robustness and frailty markers were negatively correlated (Spearman rank correlation -0.524), even without the doubly weighted item “taking a walk outside” (-0.4737).

### Predictive validity

#### Mortality

**Figure 2:**
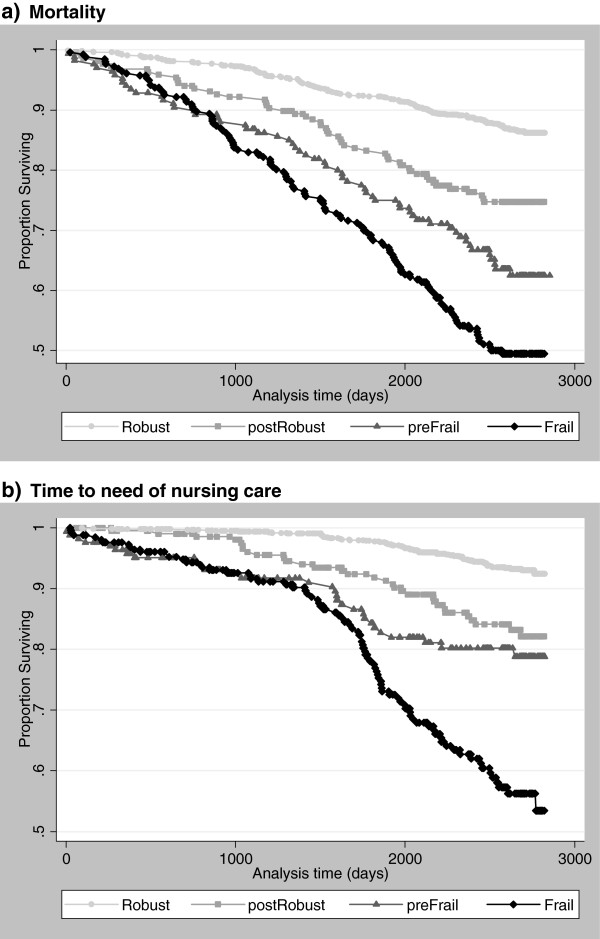
**Mortality (a) and time to need of nursing care (b) of initially A. Robust (points), B. postRobust (rectangles), C. preFrail (triangles) and D. Frail (rhombs) participants, LUCAS cohort, January 2002 – September 2009.**

Figure 2a shows the survival of the cohort participants from January 2002 to September 2009 (93 months). There were highly significant differences between the classes, with initially Robust participants surviving longest and the initially Frail shortest (log rank test = 190.2, 3 df, p < .0001). Survival differences between the FA classes remained highly significant after adjustment for sex, age and self-reported health (likelihood ratio = 22.29, 3 df, p = .0001). The FA index predicted mortality risks not explained by sex, age and self-reported health. The index was predictive over the longer term. Differences between the classes preFrail and Frail became clearly evident after almost three years.

#### Need of nursing care

There were highly significant differences in the risk of needing nursing care between the four classes (log rank test = 203.6, 3 df, p < .0001) (Figure 2b). Initially Frail participants needed care earlier than initially Robust. The differences between classes remained highly significant after adjustment for sex, age and self-reported health (likelihood ratio = 26.04, 3 df, p < .0001). The FA index was predictive over the longer term. Clear differences between the preFrail and Frail classes became evident not earlier than after four years.

Table 4 shows the loss of participants over time by cause. There were more deaths and refusals in the preFrail and Frail classes (Fisher p < .0005, p = .011), but no significant differences in losses to follow-up (Fisher p = .124). Table 5 presents selected health information six years after the initial classification. With proceeding frailty, falls, ADL dependence and the need of nursing care increased markedly (Fisher all p < .0005). At wave 2, preFrail persons were worse off than postRobust persons (excepting multiple falls), but differences appeared in some variables only (Additional file 1: Table S1).

**Table 4:** **Changes occurring between waves 1 and 2, classified by the functional ability index at wave 1**

Characteristics	ALL n = 1679 (100) cases/n (%)	Women n = 1043 (62.1) cases/n (%)	Men n = 636 (37.9) cases/n (%)	Robust n = 1022 (60.9) cases/n (%)	postRobust n = 220 (13.1) cases/n (%)	preFrail n = 172 (10.2) cases/n (%)	Transients ^*^n = 392 (23.3) cases/n (%)	Frail n = 265 (15.8) cases/n (%)
Deaths	288/1679(17.2)	159/1043(15.2)	129/636(20.3)	97/1022(9.5)	49/220(22.3)	47/172(27.3)	96/392(24.5)	95/265(35.8)
Lost to follow-up	67/1679(4.0)	53/1043(5.1)	14/636(2.2)	33/1022(3.2)	10/220(4.5)	10/172(5.8)	20/392(5.1)	14/265(5.3)
Refusals^†^	203/1679(12.1)	140/1043(13.4)	63/636(9.9)	106/1022(10.4)	21/220(9.5)	31/172(18.0)	52/392(13.3)	45/265(17.0)

*Transients: postRobust + preFrail.

^†^At wave 2; 13 missing due to missing single responses.

**Table 5:** **Selected health characteristics at wave 2, classified by the functional ability index at wave 1**

Characteristics	ALL n = 1121 (100) cases/n (%)	Women n = 691 (61.6)cases/n (%)	Men n = 430 (38.4)cases/n (%)	Robust n = 786 (70.1)cases/n (%)	postRobust n = 140 (12.5)cases/n (%)	preFrail n = 84 (7.5)cases/n (%)	Transients* n = 224 (20.0)cases/n (%)	Frail n = 111 (9.9)cases/n (%)
Falls: once^†^	177/1090(16.2)	120/669(17.9)	57/421(13.5)	104/767(13.6)	28/132(21.2)	17/82(20.7)	45/214(21.0)	28/109(25.7)
Falls: more than once^†^	114/1090(10.5)	80/669(12.0)	34/421(8.1)	53/767(6.9)	25/132(18.9)	11/82(13.4)	36/214(16.8)	25/109(22.9)
ADL dependent at wave 2^‡^	65/1102(5.9)	44/678(6.5)	21/424(5.0)	17/774(2.2)	10/135(7.4)	13/83(15.7)	23/218(10.6)	25/110(22.7)
Need of nursing care at wave 2^§^	59/1121(5.3)	38/691(5.5)	21/430(4.9)	20/786(2.5)	5/140(3.6)	9/84(10.7)	14/224(6.3)	25/111(22.5)

*Transients: postRobust + preFrail.

^†^Within one year before wave 2.

^‡^Cannot take a bath/shower without human assistance.

^§^Classified into “Pflegestufe” (German law).

### Stability and transitions between the classes robust to frail

**Figure 3:**
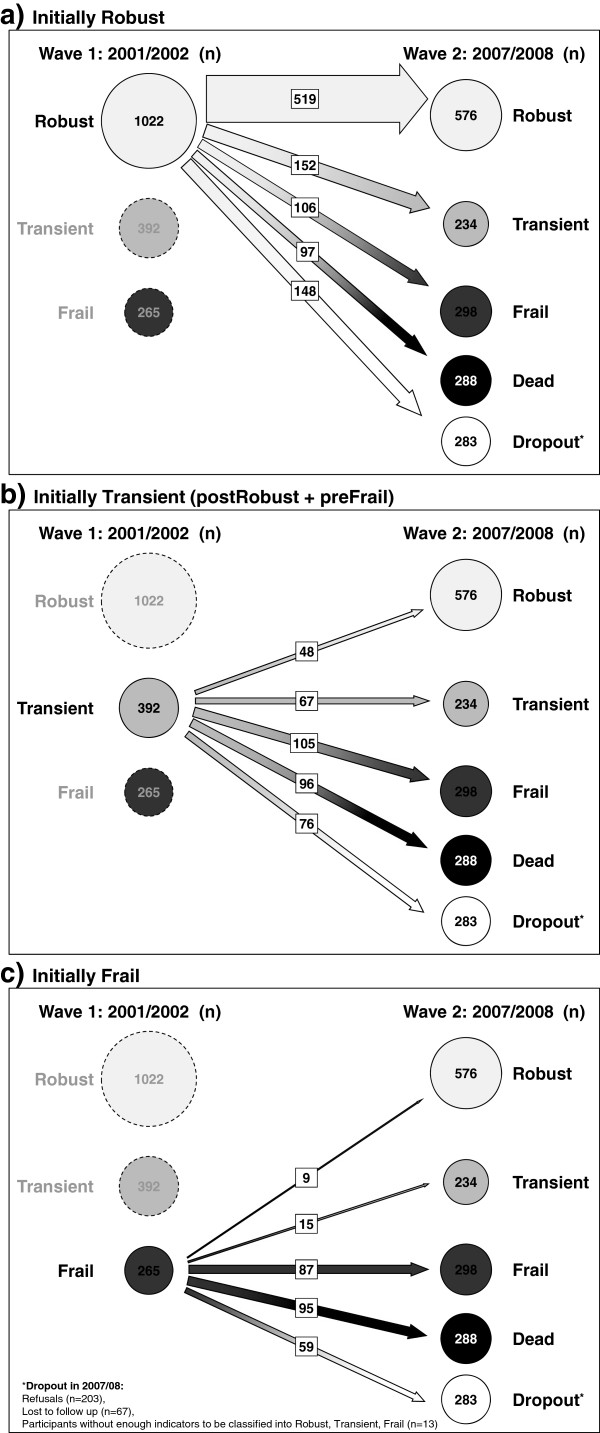
**Transitions of LUCAS participants between functional classes over six years; a)** **initially Robust, b)** **initially Transients (postRobust + preFrail)** **, c)** **initially Frail.**

We investigated transitions between the functional classes over time (Figure 3a-c). To avoid instability due to small numbers, we pooled postRobust and preFrail. Out of the 1,022 participants who were Robust at wave 1, 50.8% (519) stayed Robust six years later at wave 2, 14.9% (152) changed to Transient (postRobust + preFrail), and 10.4% (106) to Frail; 9.5% (97) died (Figure 3a). Of the 392 participants classified as Transient at wave 1, 26.8% (105) became Frail by wave 2, and 24.5% (96) had died. One eighth (12.2%; 48) moved from Transient to Robust at wave 2 (Figure 3b). Of the 265 participants initially classified as Frail, 35.8% (95) had died before wave 2. Only small fractions moved from Frail to Transient (5.7%; 15) or Robust (3.4%; 9) at wave 2 (Figure 3c).

## Discussion

Age per se is of little value to discriminate between robust, prefrail, frail and disabled community-dwelling senior citizens [30–32]. The aim of this article from the Longitudinal Urban Cohort Ageing Study (LUCAS) was to present a suitable self-administered Functional Ability (FA) index and to examine its predictive power for changes in functional status, early development of frailty, need of nursing care, and mortality over 93 months.

**Figure 4:**
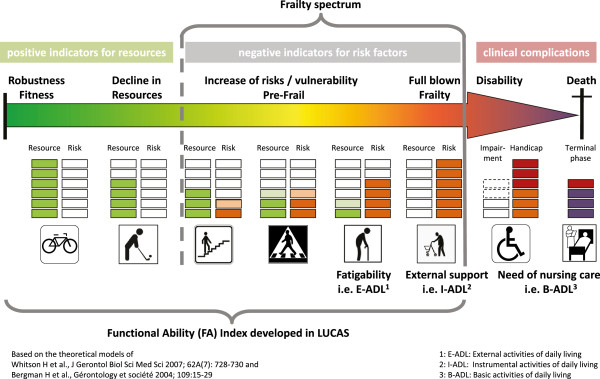
**Scheme of geriatric functional progression from independence to death underpinned with resources and risk factors in the LUCAS cohort.**

We could establish that the FA index does distinguish clearly between the classes Robust, postRobust, preFrail and Frail. These classes have different perspectives of functional decline, developing need of nursing care and survival. The FA index correlates with other health measures self-reported health, pain and depressive mood. From the public health perspective, the main finding is that the FA index discriminated between functionally independent persons (Robust), and those with grades of functional derangement (postRobust or preFrail) and functional decline (Frail), and their trajectories over a six-year period. According to the index’ outcome, individuals could profit from target group specific preventive interventions to strengthen resources and to reduce risks (Figure 4). For those persons classified as preFrail or Frail, an appropriate comprehensive assessment and geriatric intervention should follow as early as possible to counteract further functional decline.

Population-based screening in community-dwelling older people through the FA index may strengthen the shift from reactive disease management towards proactive prevention of functional decline, as recently proposed [18, 33].

Based on an earlier framework to understand the frailty process [6], the data of our study allow an individual differentiation of Frieds’ frailty phenotype [4, 11] as well as of the frailty spectrum within the geriatric functional progression [34]. Details see Figure 4. We have added resource markers, as did the Canadian study of health and aging [35], the Tilburg frailty indicator [19] and also higher-level competences as done in the TMIG [22] to further develop public health strategies to prevent frailty [36].

The FA index is distinct from multiple scales to assess frailty. All of these count deficits in health and do not concentrate with equal weight on both, health risks and health resources [37–39]. Because of this approach, the FA index is hardly comparable to other frailty indices. However, the inclusion of resources in the FA index provides the opportunity to not only distinguish between frail and non-frail persons, but also to distinguish between those persons not yet prefrail but no longer fully robust. Seniors with simultaneous accumulation of many risks and many resources (postRobust) do stay independent and live longer than seniors with few risks but also few resources at the same time (preFrail). The resource and risk criteria used in the FA index are based on self-report. The scoring can be done quickly by hand, making this index suitable for screening in GP’s practices or senior health centers, for example.

The six resources asked about do require higher performance than basic ADL (Table 1). The study shows that information on resources is important. The crucially important difference between Robust and preFrail (both with 0–2 frailty markers) as well as between postRobust and Frail (at least 3 frailty markers) is the number of resources available (Table 2). Therefore, it is meaningful to consider the importance of resources as well as their absence. See also Figure 2a-b, Table 4 shows significantly higher refusal rates between waves 1 and 2 in persons classified as preFrail compared to postRobust. We assume that with refusals at wave 2, the dwindling or lack of resources is the reason for stopping participation, often verbalized as “heavily burdened”. We are convinced of the need to consider the delicate balance of both, resources and risks in future work. In fact, our results show that risks alone do not adequately determine prefrailty or frailty.

The main methodological strength of the study is its longitudinal design, combined with the fact that the LUCAS population was initially mostly robust. A second strength is the broad range of health information available, including resources and risk factors. It appears that prefrailty and its progression can best be studied from its initial manifestations, if resources are also considered [23, 24]. In the LUCAS cohort, the FA index was predictive of survival and the time to need of nursing care over 93 months. In this initially robust population, its full discriminatory power became evident after three to five years (Figure 2a-b), indicating that the index captured functional decline at an early stage.

The transitions between functional classes suggest that the index does reflect the graduation of functional competence. Three questions explicitly addressed changes in how activities were performed, probably indicating avoidance strategies. In line with scientific evidence that the process of functional decline can be reversed [12], our graphs indicate this possibility. However, in the present report without preventive intervention, the probability of moving was higher to a less robust class than to a more robust one. The most frequent movement was to the next worse class (Figure 3a-c).

The FA index was explicitly not designed and did not identify disabled persons, the patients of established geriatric care. However, ADL dependency and any need of nursing care were quantified in every individual at each single wave of the long-term study. The propensity to develop disabilities varied markedly between the FA index classes (Table 5). Need of nursing care, the indicator we used, is identified by standardized procedures: German law defines care levels entitled for health care compensation.

A limitation of our study may be that the FA index is based on self-report data, often considered unreliable [40, 41] to identify early signs of incipient development of frailty in older people in the community. On the other hand, self-reports provide valuable information about the individuals’ perception of their own functioning in their living environment [42]. Second, the three questions on changes in activity performance might cause problems, as change may have occurred too long ago to be reported. However, an extended clinical assessment in a 10% random sample of the LUCAS cohort confirmed the functional grading by the FA index [30]. Finally, LUCAS as other cohort studies had refusals and lost participants. Both, refusals and losses to follow-up occurred more frequently in preFrail and Frail participants (Table 4). In future practice, non-responders are indicated as due for appropriate assessment.

In summary, the Functional Ability (FA) index may increase the awareness of (pre)frailty and consequences: risk of disability, hospitalization, dependency, and mortality. This is in accordance to the “frailty consensus: a call to action” [43] and of relevance to health-care providers and the public, in general.

## Conclusions

1. The FA index questions about daily-life habits help to identify early signs of functional decline, becoming frail and disabled, and signal where to initiate appropriate preventive measures.
2. The FA index can be used to operationalize the terms Robust, postRobust, preFrail and Frail, and thereby to initiate target group specific interventions.
3. Consistently over time, the FA index correlated significantly with self-reported health, depressive mood and chronic pain.
4. The FA index was predictive of the need of daily nursing care and mortality. Its prognostic validity was established in a large longitudinal cohort study.

The FA index as described in this article provides an easy way to screen the heterogeneous population of community-dwelling senior citizens for early signs of functional decline by focussing equally on both, a decline in resources and an increase of risk factors. The questionnaire can be completed within a few minutes at home or before a visit to the doctor, and its classification can be quickly done by hand, i.e. in general practitioner practices. We expect that this will increase the awareness of physicians, public health professionals and health planners of needs in the primary care sector and the rapidly ageing population regarding early prevention of functional decline.

## Electronic supplementary material

Additional file 1: Table S1: Responses to FRAIL and ROBUST marker questions at 2001/2002 (LUCAS cohort, wave 1). (DOC 46 KB)
