# Supplementary material for: Long-term prediction of changes in health status, frailty, nursing care and mortality in community-dwelling senior citizens - results from the longitudinal urban cohort ageing study (LUCAS)
Source: BMC Geriatr. 2014 Dec 19;14:141. doi: 10.1186/1471-2318-14-141 (PMC4289576; doi:10.1186/1471-2318-14-141)
Supplement: Supplementary file 1 — Additional file 1: Table S1: Responses to FRAIL and ROBUST marker questions at 2001/2002 (LUCAS cohort, wave 1). (DOC 46 KB) [file 12877_2014_1074_MOESM1_ESM.doc]

**Additional file 1: Table S1. Responses to FRAIL and ROBUST marker questions at 2001/2002 (LUCAS cohort, wave 1)**

**Exact question wordings see Table 1**

| **Responses at wave 1**  **(2001/2002)** | **ALL**  **n=1679**  **Cases/n (%)** | **Women**  **n=1043**  **Cases/n (%)** | **Men**  **n=636**  **Cases/n (%)** | **Robust**  **n=1022**  **Cases/n (%)** | **postRobust^*^**  **n=220**  **Cases/n (%)** | **preFrail^*^**  **n=172**  **Cases/n (%)** | **Transients^*^**  **n=392**  **Cases/n (%)** | **Frail**  **n=265**  **Cases/n (%)** |
| --- | --- | --- | --- | --- | --- | --- | --- | --- |
| FRAIL 1: unexpected weight loss  over the past 6 months – yes | 90/1660  (5.4) | 62/1030  (6.0) | 28/630  (4.4) | 25/1013  (2.5) | 29/216  (13.4) | 6/169  (3.6) | 35/385  (9.1) | 30/262  (11.5) |
| FRAIL 2: changed the way to walk 1  kilometer in the past 12 months – yes | 548/1645  (33.3) | 360/1012  (35.6) | 188/633  (29.7) | 95/1002  (9.5) | 193/219  (88.1) | 38/161  (23.6) | 231/380  (60.8) | 222/263  (84.4) |
| FRAIL 3: changed the way to climb 10  steps in the past 12 months – yes | 713/1653  (43.1) | 476/1022  (46.6) | 237/631  (37.6) | 184/1005  (18.3) | 215/218  (98.6) | 58/165  (35.2) | 273/383  (71.3) | 256/265  (96.6) |
| FRAIL 4: changed the way to get into/out  of car/bus in the past 12 months – yes | 544/1650  (33.0) | 393/1020  (38.5) | 151/630  (24.0) | 79/1004  (7.9) | 185/218  (84.9) | 37/164  (22.6) | 222/382  (58.1) | 243/264  (92.0) |
| FRAIL 5: walking outside –  never/1-2 days per week | 212/1632  (13.0) | 132/1003  (13.2) | 80/629  (12.7) | 46/1011  (4.6) | 28/218  (12.8) | 49/149  (32.9) | 77/367  (21.0) | 89/254  (35.0) |
| FRAIL 6: fallen ever during the past  12 months – yes | 404/1589  (25.4) | 290/986  (29.4) | 114/603  (18.9) | 155/979  (15.8) | 87/211  (41.2) | 32/151  (21.2) | 119/362  (32.9) | 130/248  (52.4) |
| ROBUST 1: activity performance: able  to walk 500 meters – yes | 1394/1654  (84.3) | 852/1027  (83.0) | 542/627  (86.4) | 1000/1020  (98.0) | 197/218  (90.4) | 108/162  (66.7) | 305/380  (80.3) | 89/254  (35.0) |
| ROBUST 2: walking outside over the  past 7 days – 3-4 / 5-7 days per week | 1420/1632  (87.0) | 871/1003  (86.8) | 549/629  (87.3) | 965/1011  (95.5) | 190/218  (87.2) | 100/149  (67.1) | 290/367  (79.0) | 165/254  (65.0) |
| ROBUST 3: moderate exercises over  the past 7 days – 1-7 days per week | 968/1592  (60.8) | 595/975  (61.0) | 373/617  (60.5) | 743/1001  (74.2) | 163/215  (75.8) | 15/139  (10.8) | 178/354  (50.3) | 47/237  (19.8) |
| ROBUST 4: intense exercises over the  past 7 days – 1-7 days per week | 255/1529  (16.7) | 133/933  (14.3) | 122/596  (20.5) | 217/962  (22.6) | 36/209  (17.2) | 0/172  (0) | 36/343  (10.5) | 2/224  (0.9) |
| ROBUST 5: socially engaged: work  as a volunteer – yes | 260/1608  (16.2) | 131/987  (13.3) | 129/621  (20.8) | 216/989  (21.8) | 38/215  (17.7) | 1/156  (0.6) | 39/371  (10.5) | 5/248  (2.0) |
| ROBUST 6: limitation of activities  due to fear of falling – no | 1208/1620  (74.6) | 687/1002  (68.6) | 521/618  (84.3) | 938/1008  (93.1) | 163/215  (75.8) | 51/149  (34.2) | 214/364  (58.8) | 56/248  (22.6) |

* postRobust: those persons showing simultaneously characteristics of robustness (3-6 resources) and frailty (3-6 risks); preFrail: those persons showing neither characteristics of robustness (0-2 resources) nor frailty (0-2 risks); Transients: postRobust + preFrail
